# Micromolar fluoride contamination arising from glass NMR tubes and a simple solution for biomolecular applications

**DOI:** 10.1007/s10858-024-00442-x

**Published:** 2024-07-27

**Authors:** Khushboo Matwani, Jasmine Cornish, Erika Alden DeBenedictis, Gabriella T. Heller

**Affiliations:** 1 Department of Structural and Molecular Biology, Division of Biosciences, University College London, London, WC1E 6BT UK; 2 The Francis Crick Institute, London, NW1 1AT UK

**Keywords:** ^19^F NMR, Fluoride, Tubes, Glass, Quartz, Contamination

## Abstract

Fluorine (^19^F) NMR is emerging as an invaluable analytical technique in chemistry, biochemistry, structural biology, material science, drug discovery, and medicine, especially due to the inherent rarity of naturally occurring fluorine in biological, organic, and inorganic compounds. Here, we revisit the under-reported problem of fluoride leaching from new and unused glass NMR tubes. We characterised the leaching of free fluoride from various types of new and unused glass NMR tubes over the course of several hours and quantify this contaminant to be at micromolar concentrations for typical NMR sample volumes across multiple glass types and brands. We find that this artefact is undetectable for samples prepared in quartz NMR tubes within the timeframes of our experiments. We also observed that pre-soaking new glass NMR tubes combined with rinsing removes this contamination below micromolar levels. Given the increasing popularity of ^19^F NMR across a wide range of fields, increasing popularity of single-use screening tubes, the long collection times required for relaxation studies and samples of low concentrations, and the importance of avoiding contamination in all NMR experiments, we anticipate that our simple solution will be useful to biomolecular NMR spectroscopists.

## Introduction

Over the past seven decades, fluorine (^19^F) NMR has emerged as an invaluable analytical technique for a wide range of scientific applications, including chemistry, biochemistry (Aramini et al. 2014; Boeszoermenyi et al. 2020; Gronenborn 2022; Kim et al. 2013; Lu et al. 2019; Picard and Prosser 2021), material science (Nartowski et al. 2016; Xu et al. 2017), medicine (Ojima 2009; Ruiz-Cabello et al. 2011), structural biology (Li et al. 2010; Pham et al. 2023; Zhu et al. 2022), and drug discovery (Dalvit et al. 2020; Rüdisser et al. 2020). A distinguishing feature of ^19^F NMR, in addition to the remarkable sensitivity of the ^19^F nucleus, is the inherent rarity of naturally occurring fluorine in biological, organic, and inorganic compounds. Biochemically, ^19^F probes can be artificially introduced into proteins via post-translational modifications (Brauer and Sykes 1986) or via incorporation of fluorinated amino acids into growth media (Crowley et al. 2012). Fluorine can also be included within small molecules: to date, approximately 50% of all marketed agrochemicals (Bégué and Bonnet-Delpon 2005), and more than 30% of recent FDA-approved pharmaceuticals contain ^19^F (Chandra et al. 2023; Han et al. 2020; Müller et al. 2007). Consequently, background interference, which is a common concern in other NMR nuclei, should, in principle, be virtually non-existent in the context of ^19^F NMR.

Fluorine chemists are likely familiar with the fact that fluorine is used in the refinement process of glass and consequently, fluoride ions leach out from glass NMR tubes potentially confounding the analysis of ^19^F spectra. Given the expansion of applications of ^19^F NMR, the increasing popularity of ^19^F NMR in aqueous solutions (Li et al. 2010; Pham et al. 2023; Zhu et al. 2022; Dalvit et al. 2020; Rüdisser et al. 2020), and the improved sensitivity of NMR instruments over the past few decades, we have quantified and characterised these fluoride artefacts and suggest a simple, cost-effective solution that can be implemented when using new, glass NMR tubes.

## Results

**Fig. 1:**
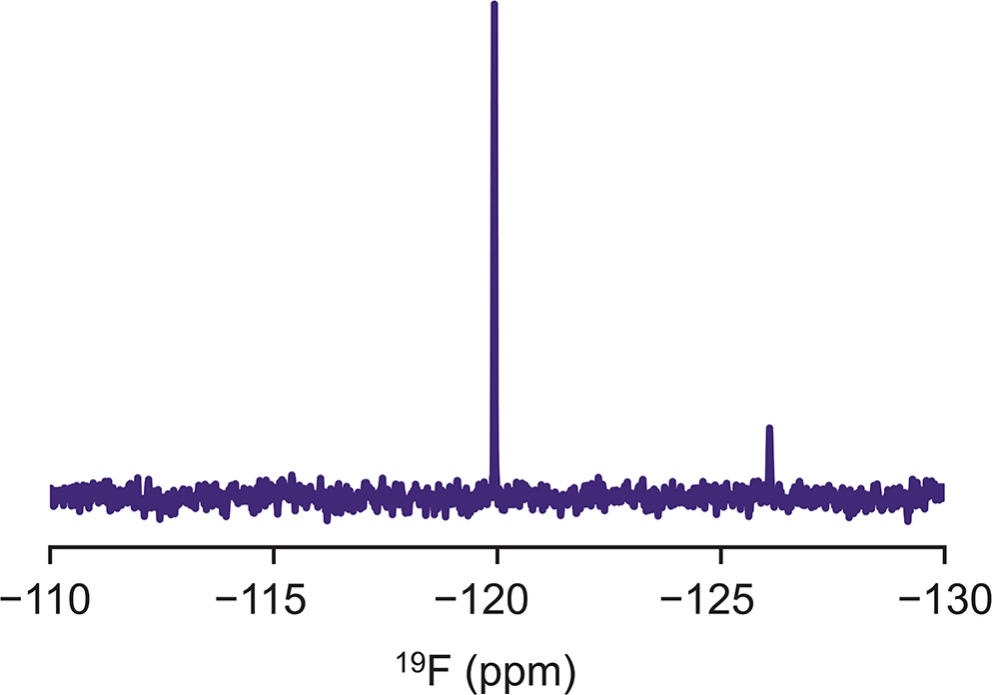
^19^F NMR spectrum of 20 μM 5-fluoroindole in PBS buffer, showing the expected peak for 5-fluoroindole near − 126 ppm and the fluoride peak near − 120 ppm

**Fig. 2:**
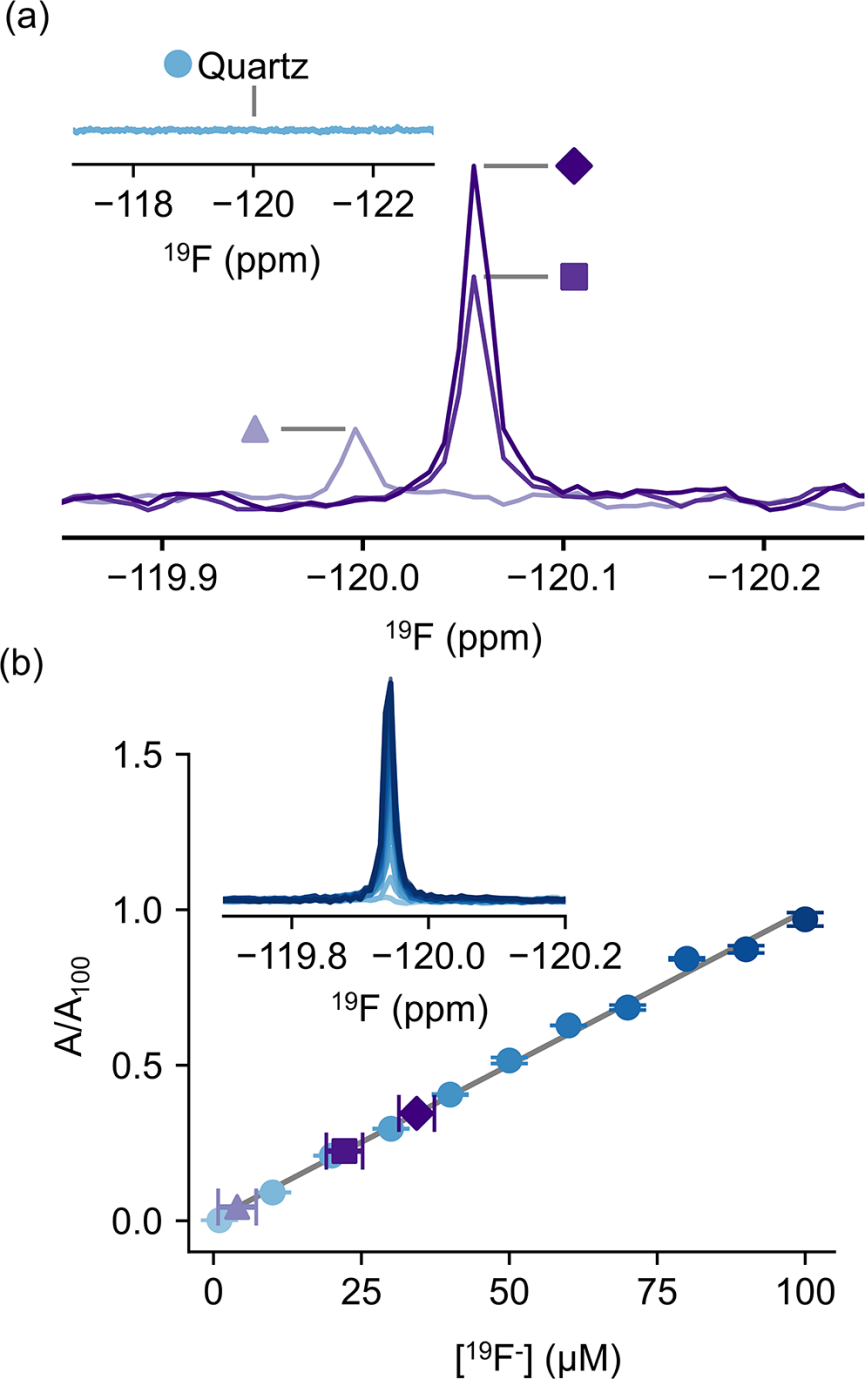
The fluoride peak near − 120 ppm is observed in samples containing buffer alone in three types of glass NMR tubes, but not in quartz tubes. (**a**) ^19^F spectra of PBS incubated in various types of new, unused glass NMR tubes for 48 h. Triangle: Wilmad Precision, type I, class A; square: Wilmad High Throughput, type I, class B; diamond: Norell Secure, type I, class B. Insert shows ^19^F spectra of PBS incubated in a new quartz tube for 48 h. (**b**) Quantification of unknown fluoride concentrations in various glass tubes. Insert shows ^19^F spectra of known fluoride standards measured in quartz tubes. Normalised peak area is plotted as a function of fluoride concentration to determine fluoride quantities in various tube types. Triangle: Wilmad Precision, type I, class A (4 ± 3 μM); square: Wilmad High Throughput, type I, class B (22 ± 3 μM); diamond: Norell Secure, type I, class B (34 ± 3 μM). Y-error bars represent one SD and x-error bars represent the prediction interval

**Fig. 3:**
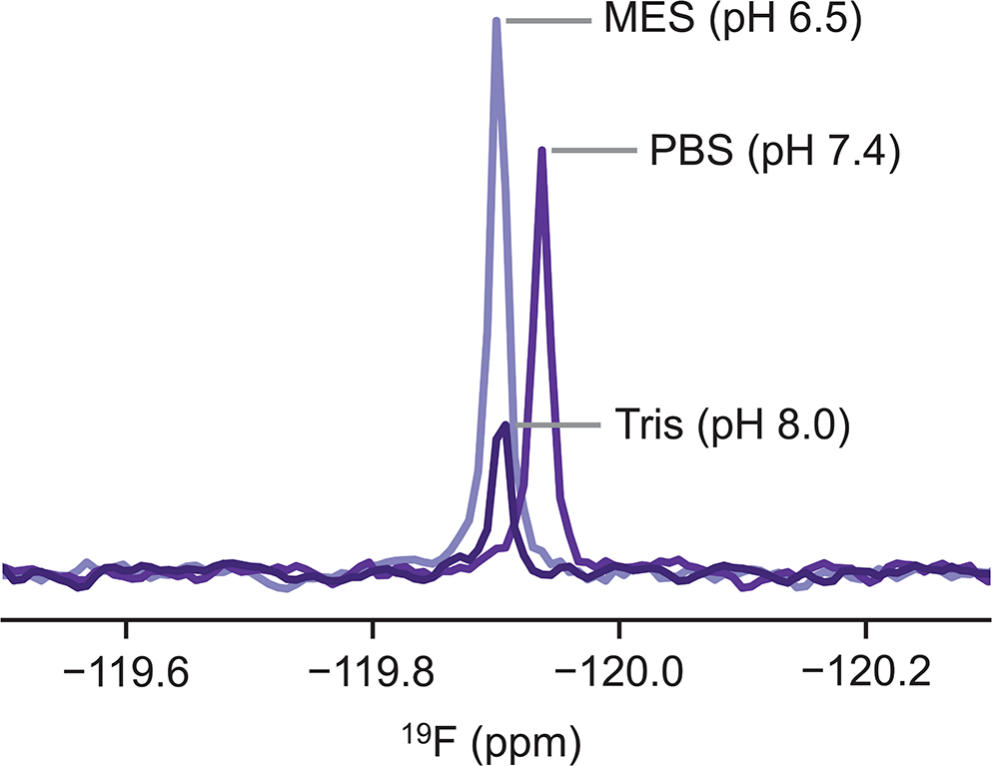
The fluoride peak is observed in various buffers of diverse pH values after incubation in glass tubes (type I, class B) for 48 h

To demonstrate the potential confusion caused by free fluoride in NMR analyses, we performed routine ^19^F NMR experiments of 5-fluoroindole (with an expected ^19^F chemical shift near − 126 ppm) in phosphate buffered saline (PBS) using a new, glass NMR tube. In addition to the expected peak from 5-fluoroindole, we observed a second sharp peak near − 120 ppm, corresponding to free fluoride (Fig. 1). This peak appeared in spectra measured of solutions containing buffer alone across several brands, types, and classes of new 5 mm NMR tubes (Fig. 2), including Wilmad Precision (type I, class A), Wilmad High Throughput (type I, class B), and Norell Secure (type I, class B) tubes. The fluoride contamination was also present to varying degrees across different buffers (Fig. 3), but undetectable within the measurement time for samples prepared in quartz tubes (Wilmad), suggesting the source of the contamination is the glass tube itself (Fig. 2a).

To quantify the concentration of the fluoride contaminant, we prepared control samples of sodium fluoride in PBS in pre-rinsed quartz tubes and observed peaks of a similar nature to the artefact (e.g. chemical shift, peak widths). Using sodium fluoride standards ranging from 1 to 100 μM in pre-rinsed quartz tubes, we quantified the concentrations of free fluoride leaching from the various glass tubes (Fig. 2b). We quantified free fluoride to be at micromolar concentrations after 48 h of incubation for all glass tubes; we approximate the concentrations of free fluoride to be 34 ± 3 μM for Norell Secure (type I, class B) tubes, 22 ± 3 μM for Wilmad High Throughput (type I, class B), and 4 ± 3 μM for Wilmad Precision (type I, class A) tubes.

**Fig. 4:**
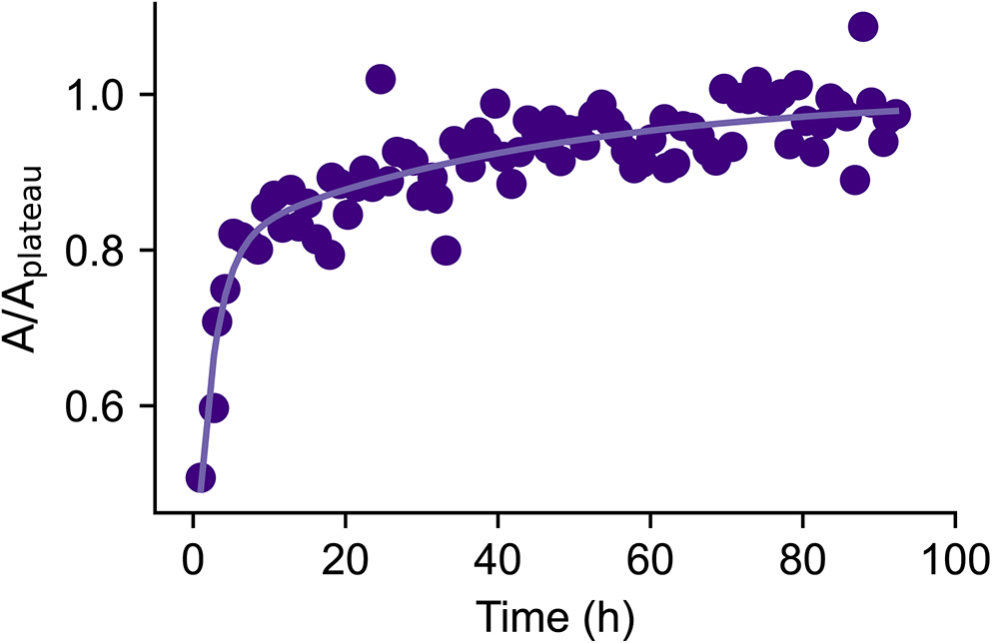
The release of free fluoride from glass NMR tubes over time. A freshly made sample of PBS was incubated in a new, unused glass NMR tube (type I, class B) and data was collected approximately every hour. The normalised integral of the growing peak over time was fit using a bi-exponential function (see Methods)

To probe the time dependence of fluoride leaching, we monitored fluoride signal intensities in ^19^F NMR spectra. Within an hour of placing buffer in a new Norell Secure (type I, class B) NMR tube, we collected spectra approximately every hour for 92 h (Fig. 4). We observed a steep increase within the first 10 h, and with the fluoride content eventually tapering off to a plateau value corresponding to approximately 31 μM (Fig. 4).

**Fig. 5:**
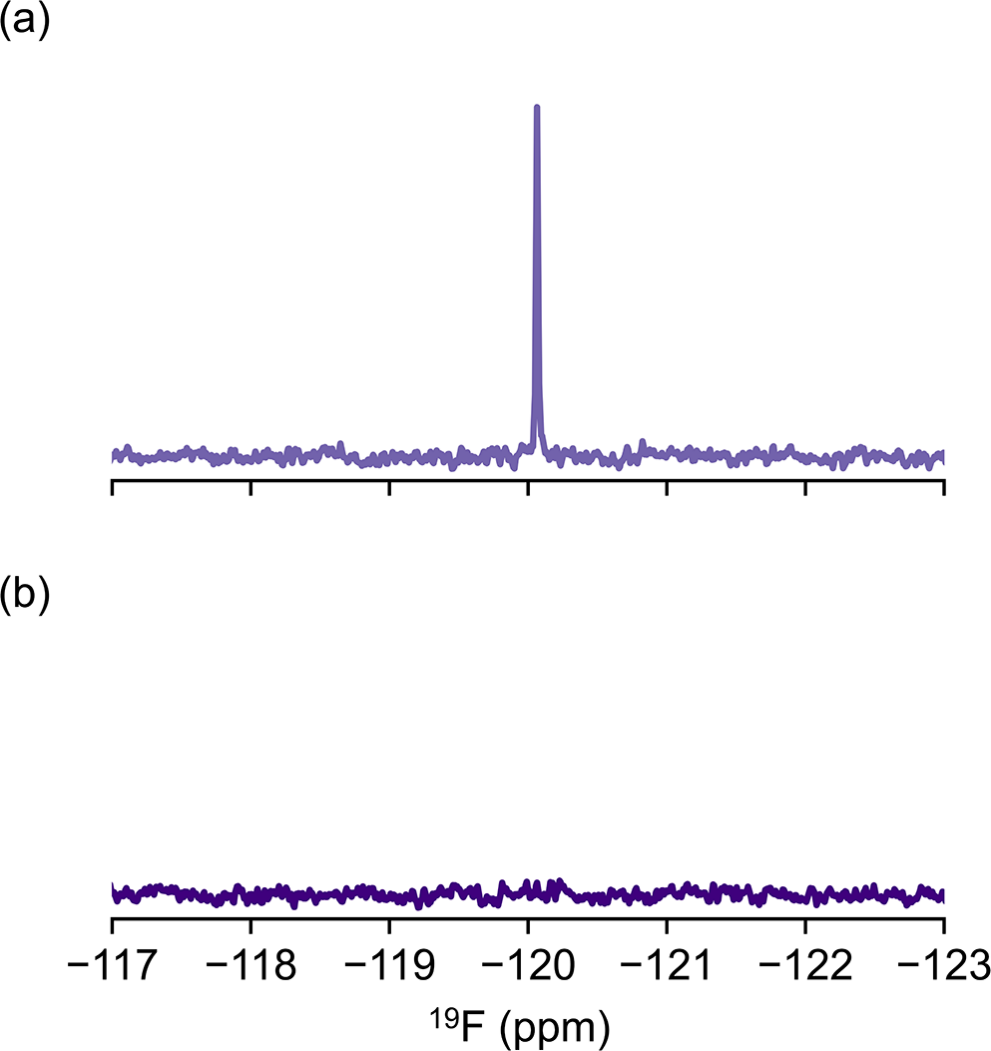
Pre-soaking NMR tubes with buffer is an easy strategy to eliminate the fluoride contaminant from glass. (**a**) ^19^F NMR spectrum collected of PBS buffer in a new glass NMR tube (type I, class B) which had been incubated with buffer for 48 h. (**b**) The sample from (a) was removed from the NMR tube, rinsed once with water and once with buffer before being incubated again with PBS. The ^19^F NMR spectrum collected after the rinse suggests the fluoride concentration is below 1 μM

Biomolecular NMR experiments, particularly relaxation studies or measurements of samples containing low concentrations, can often take several hours, and commonly samples are stored for several days or longer between measurements. We therefore wondered whether extended incubation and subsequent rinsing of the tube could remove the fluoride contamination. To test this, we incubated a Norell Standard (type I, class B) tube (the same glass type as the Norell Secure tube) in PBS for 48 h. As we predicted, significant leaching of fluoride ions was observed. We then rinsed the tube twice (once with water and once with buffer) and recollected the ^19^F NMR spectrum. The absence of any peak near − 120 ppm suggests that the fluoride level was below 1 μM (Fig. 5).

**Fig. 6:**
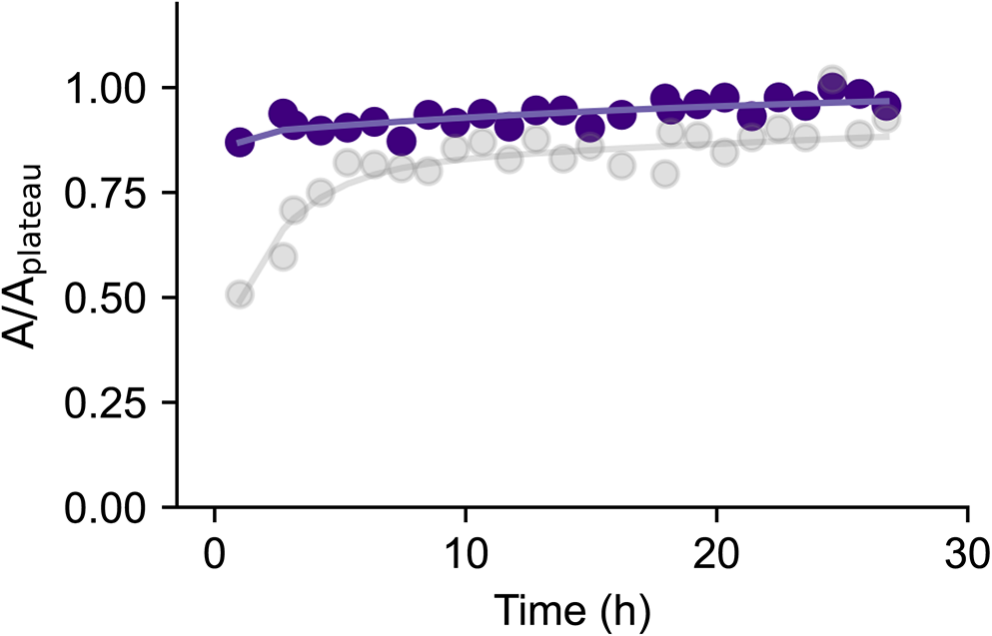
Fluoride leaching is accelerated at a higher temperature. A sample of PBS was incubated in a new, unused glass NMR tube (type I, class B) at 60 °C (violet) and 25 °C (grey, Fig. 4). Data was collected approximately every hour. The integral of the peak over time was normalised and fit using a bi-exponential function (see Methods)

Given that the fluoride leaching process takes more than a day to plateau, we wondered whether we could accelerate the leaching process for faster removal of free fluoride. To this end, we incubated a Norell Standard (type I, class B) tube with PBS buffer at 60 °C, and monitored the fluoride peak over time. We immediately observed the appearance of the fluoride peak within an hour and that the intensity of this peak plateaus soon thereafter (Fig. 6).

**Fig. 7:**
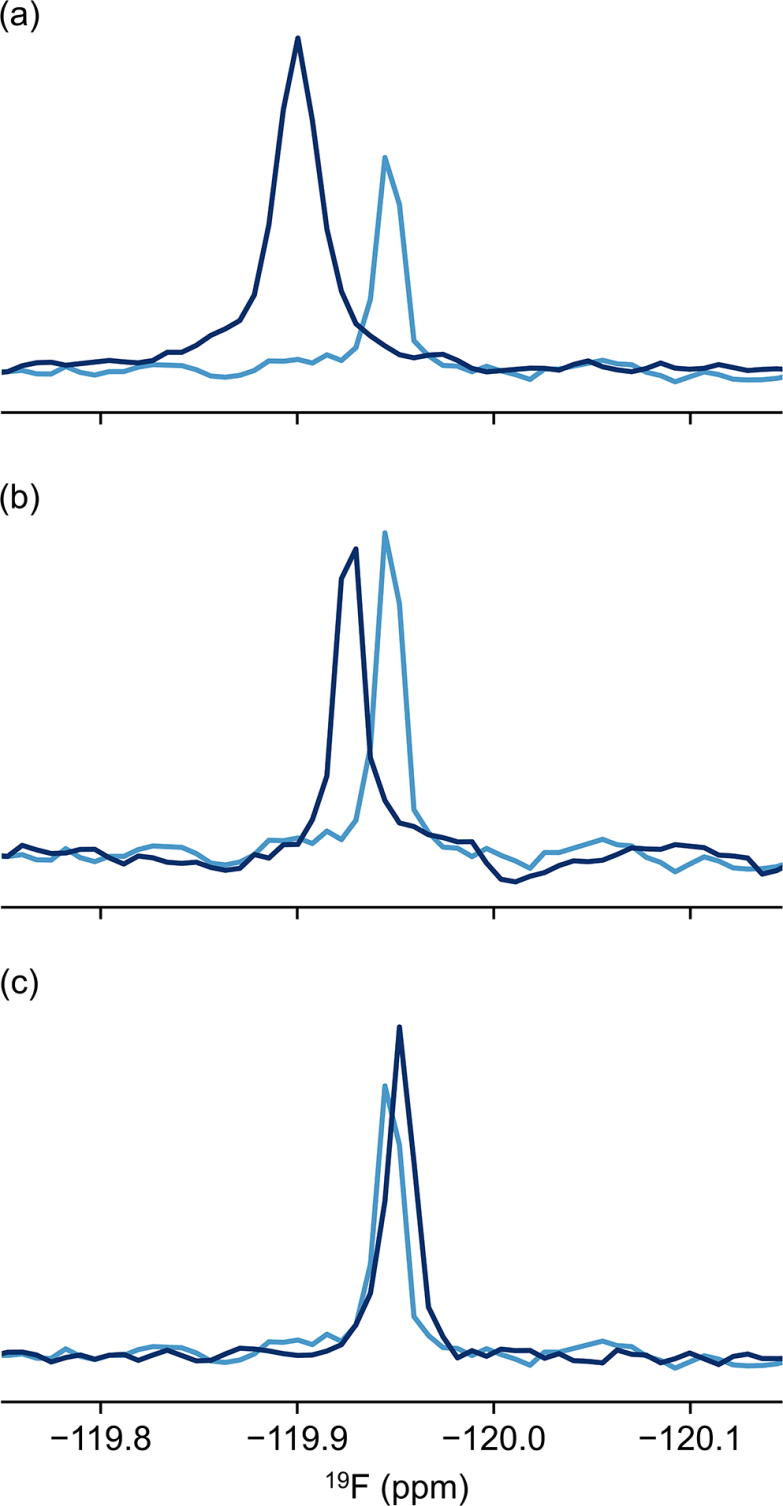
Chemical shift perturbations of fluoride suggest that the ion binds various proteins. ^19^F NMR spectra of a 20 μM sodium fluoride sample in the absence (light blue) and presence of various proteins (dark blue) including 100 μM DNase (**a**), 200 μM BSA (**b**), and 107 μM NS5A D2-D3 (**c**)

To investigate the interference of fluoride with biomolecular samples, we incubated known concentrations of sodium fluoride with various proteins. We observed that free fluoride, at the contamination concentrations reported herein, binds to various proteins including DNase (Fig. 7a), bovine serum albumin (Ayranci and Duman 2004) (BSA, Fig. 7b), and the intrinsically disordered domains 2 and 3 of the non-structural protein 5 A (NS5A-D2D3, Fig. 7c) from hepatitis C virus, indicated by a ^19^F chemical shift perturbation of the fluoride ion.

## Discussion and conclusions

Here, we characterise free fluoride leaching from NMR glass tubes. The chemical shift of this peak is in close proximity to and could easily be mistaken for peaks of biochemical and/or medicinal chemistry interest, for example ^19^F-labelled tryptophan residues (labelled with 5-fluoroindole) which often occur in range − 123 to −126 ppm (Gimenez et al. 2021), and several fluoro-aromatic compounds (for example in the context of ^19^F small-molecule screening) which often fall in the range between − 60 and − 172 ppm (Dolbier 2016; Jang and Kim 2020; Saunders et al. 2018).

We noted that this phenomenon is undetectable in quartz tubes. Quartz has a modular molecular structure formed of highly regular tetrahedral SiO_2_ units, with minimal impurities (Anas Boussaa et al. 2016; Götze et al. 2021), potentially explaining why we were unable to detect fluoride. Borosilicate glass on the other hand, from which typical glass NMR tubes are made, is comprised of an amorphous structure consisting of boron atoms in a tetrahedral or trigonal conformation with silicon and bridging oxygen atoms (Hasanuzzaman et al. 2016). Fluorides are introduced during the refinement process, acting as a nucleating agent which increases the durability of glass (Ceriani et al. 1985; Varshneya and Mauro 2019). Fluorides are also used as additives to further modify the glass network, increasing the pore size of the network formation (Takusagawa et al. 1987). Fluoride (De Maeyer et al. 1999; Elbatal et al. 2016; Moynihan and Loehr 1988), and other ions (Bunker et al. 1986; Bunker and Casey 2016; Frankel et al. 2018), have been shown to leach from different glass types over time. The dissolution process can take place over the span of hours and days as demonstrated in other studies (Bunker et al. 1986).

Fluoride leaching from glass has been previously reported for other glass surfaces (De Maeyer et al. 1999; Elbatal et al. 2016; Moynihan and Loehr 1988) and this is particularly relevant in the context of typical NMR tubes, as they have a relatively high surface area/volume ratio. Here, we report that various types and brands of new, unused glass NMR tubes can lead to micromolar contaminations of fluoride, comparable to the concentrations used in many biochemical and drug-discovery experiments, which can significantly complicate NMR analyses. In the context of biochemistry, fluoride has been shown to inhibit enzymes such as enolases (Qin et al. 2006) and hydrolases (Guranowski 1990), bind proteins, and is essential for the function of nucleic acid structures like riboswitches (Maxwell et al. 2023). While using quartz tubes is one simple way to minimise fluoride contamination, quartz tubes can cost up to 7.5 times more than high-field glass tubes. As the fluoride concentration plateaus within 20 h at room temperature, and even sooner at 60 °C, incubating new glass tubes at 60 °C followed by a rinse can be a time- and cost-effective, one-off strategy to minimise fluoride contamination.

Artefacts arising from long incubation times are particularly relevant for dynamic studies (e.g. Carr-Purcell-Meiboom-Gill and Chemical Exchange Saturation Transfer experiments) (Overbeck et al. 2020) which can last from hours to days. In the context of ^19^F drug screening, the fluoride signal may be confused for signals arising from small molecules (e.g. fluoroaromatics), contaminants, impurities, or may further complicate interaction studies (Heller et al. 2024). While our study is limited to only a few sample conditions, we anticipate that this simple, inexpensive solution for removing fluoride contaminations by pre-soaking tubes will improve the quality of not only ^19^F spectra, but also of all biomolecular NMR data containing molecules which are sensitive to fluoride.

## Materials and methods

### Sample preparation

Phosphate buffered saline (PBS) buffer (pH 7.4) was prepared by dissolving PBS tablets (Dulbecco A, Oxoid) in water according to the manufacturer’s instructions. 2-(N-morpholino)ethanesulfonic acid (MES) buffer was made at 25 mM with 140 mM NaCl and the pH was adjusted to 6.5. Tris(hydroxymethyl)aminomethane (tris) buffer was made at 25mM with 140 mM NaCl and the pH was adjusted to 8.0.

A recombinant construct of the D2 and D3 domains of HCV NS5A (NS5A-D2D3, residues 247–466) was expressed and purified as previously described (Heller et al. 2024). NS5A-D2D3 was prepared in PBS with 1 mM tris(2-carboxyethyl)phosphine (TCEP), pH 7.4. The protein was concentrated using an Amicon Ultra Centrifugal filter with a 3 kDa cutoff.

DNAse I (Roche) and BSA (Sigma-Aldrich) were made up to 1 mM in PBS with 1 mM TCEP, pH 7.4, and then diluted to 100 μM and 200 μM respectively.

5-fluoroindole (Sigma-Aldrich) was dissolved in DMSO-d_6_ at 1 M concentration and kept frozen at -20 °C until use.

Samples of PBS buffer and 2% D_2_O were prepared to a final volume of 550 μL loaded into three different types of new 5 mm NMR glass tubes: Norell Secure (type I, class B), Wilmad Thin Wall Precision (type I, class A), and Wilmad High Throughput (type I, class B) and incubated for over 48 h. MES and tris buffers with 2% D_2_O were prepared to a final volume of 550 μL, loaded into new 5 mm Norell Standard NMR tubes (type I, class B) and incubated over 48 h. As a control, PBS buffer was incubated for the same time period in a new quartz tube (Wilmad).

Sodium fluoride standards were prepared in PBS buffer (pH 7.4) at concentrations ranging from 1 μM to 100 μM and measured in pre-rinsed quartz tubes.

### NMR experiments

^19^F spectra were collected on a 18.8 T Bruker Avance III 800 MHz spectrometer equipped with a TCI cryoprobe at 298–333 K. ^19^F spectra were acquired using the standard aring Bruker pulse sequence to minimise acoustic ringing. Spectra were obtained with 8,192 complex points, a spectral width of 45,455 Hz, a relaxation delay of 0.5 s, and an acquisition time of 0.09 s. 6,400 scans were collected per experiment, and the offset was set to -120.00 ppm. ^19^F chemical shifts were indirectly referenced with respect to trichlorofluoromethane (CFCl_3_) through the lock signal. Data was processed and analysed using nmrPipe (Delaglio et al. 1995) and nmrGlue (Helmus and Jaroniec 2013). ^19^F peaks were fit to Lorentzian curves.

Peak integrals of known fluoride standards were calculated and plotted as a function of fluoride concentration. A linear fit of the data was used to calculate unknown fluoride concentrations in various glass tubes.

To investigate the kinetics of the leaching process, a sample was prepared as described above and added to a Norell Secure tube (type I, class B). Data collection began within one hour of placing the sample in the NMR tube. Peak integrals were fit to the following bi-exponential curve:

$$\left[{{}^{19}\text{F}}^{-}\right]= A (1-B{e}^{-{k}_{1}t}-C{e}^{-{k}_{2}t})$$

where $A$ is the plateau of the curve, $t$ represents time, and $B$, $C$, ${k}_{1}$ and ${k}_{2}$ are constants.

Free fluoride was removed from a Norell Standard NMR tube (type I, class B) by pre-soaking for 48 h with PBS buffer. The tube was then rinsed twice, once with water and once with PBS before a fresh sample was added, and a spectrum was collected.

## Data Availability

Data and analysis scripts that support the findings of this study are provided here: https://github.com/gthh2/fluoride_contamination.
